# Simultaneous Multi-Treatment Strategy for Brain Tumor Reduction via Nonlinear Control

**DOI:** 10.3390/brainsci15020207

**Published:** 2025-02-17

**Authors:** Muhammad Arsalan, Xiaojun Yu, Muhammad Tariq Sadiq, Ahmad Almogren

**Affiliations:** 1 School of Automation, Northwestern Polytechnical University, Xi’an 710072, China; m_arsalan@mail.nwpu.edu.cn (M.A.); xjyu@nwpu.edu.cn (X.Y.); 2 School of Computer Science and Electronic Engineering, University of Essex, Colchester CO4 3SQ, UK; 3 Department of Computer Science, College of Computer and Information Sciences, King Saud University, Riyadh 11633, Saudi Arabia; ahalmogren@ksu.edu.sa

**Keywords:** radiotherapy, chemotherapy, nonlinear control, brain tumor mitigation, synergetic control

## Abstract

**Background**: Recently proposed brain-tumor treatment strategies prioritize fast reduction of tumor cell population while often neglecting the radiation or chemotherapeutic drug dosage requirements to achieve it. Moreover, these techniques provide chemotherapy based treatment strategies, while ignoring the toxic side effects of the drugs employed by it. **Methods**: This study updates the recently proposed brain-tumor system dynamics by incorporating radiotherapy along with chemotherapy to simultaneously initiate both therapies for a more comprehensive and effective response against tumor proliferation. Afterwards, based on the upgraded system dynamics, this study proposes a novel multi-input sigmoid-based smooth synergetic nonlinear controller with the aim to reduce the dosage requirements of both therapies while keeping the overall system response robust and efficient. The novelty of this study lies in the combination of radiotherapy and chemotherapy inputs in a way that prioritizes patients health and well-being, while integrating advanced synergetic control technique with a sigmoid function based smoothing agent. **Results**: The proposed method reduced baseline radiation and chemo drug dosages by 57% and 33% respectively while effectively suppressing tumor growth and proliferation. Similarly, the proposed controller reduced the time required for complete tumor mitigation by 60% while reducing the radiation and chemotherapeutic drug intensity by 93.8% and 21.3% respectively. **Conclusions**: This study offers significant improvement in tumor treatment methodologies by providing a safer, less riskier brain-tumor treatment strategy that has promising potential to improve survival rates against this menacing health condition so that the affected patients may lead a healthier and better quality of life.

## 1. Introduction

A brain tumor is a menacing ailment, characterized by the formulation of an anomalous tissue mass due to unrestrained cell division and an abnormal growth of unhealthy brain cells [1]. Tumors are normally categorized as malignant (cancerous) or benign (not-cancerous). Malignant tumors may spread to other tissues whereas benign tumors do not spread to other parts of body [2]. Around 20 million diagnosed cases of cancer were recorded in 2022, and this figure is expected to reach around 35 million by 2050. Additionally, the number of cancer related deaths has soared to 9.7 million [3,4]. Timely diagnosis and prompt initiation of anti-tumor procedure increase both the chances of survival as well as the life expectancy of the diseased person [5]. At present, chemotherapy, radiotherapy, hormone therapy, immuno-therapy, anti-angiogenic therapy, targeted therapy, monoclonal antibody therapy, and tissue removal through surgery are the proven treatment options against tumor spread [6,7,8,9].

Clinically, a structured approach based on a combination of multiple treatment procedures such as surgery, chemotherapy, and radiation therapy is opted for nowadays. The treatment procedure depends on tumor type, grade and patient-specific factors [10]. Currently, the following tumor treatment regimens are considered generally:

1. First-Line Treatment:In clinical practice, high-grade tumors (with poorly differentiated, abnormal looking, disorganized but fast-spreading tumor cells) are initially removed through surgery, followed by concurrent radiotherapy and chemotherapy based on temozolomide (TMZ). In contrast, low-grade tumors necessitate careful observation after surgery and depending on tumor progression, the treatment may or may not involve delayed radiation and chemotherapy (the Stupp protocol) [11,12].
2. Second-Line Treatment: In the event of the failure of first-line treatment or the development of some intolerable side effects, second-line treatment is employed. In case of recurring tumors, the approved drug Bevacizumab (vascular endothelial growth factor inhibitor) is utilized, often with chemotherapy or re-irradiation [13]. Similarly, patients are also enrolled in clinical trials for novel targeted therapies, immuno-therapy, or other experimental treatments to cure dynamically adapting tumors [14].
3. Linear Treatment Framework: These treatment procedures generally observe a linear pattern of fixed drug-dosage at fixed intervals. Clinical evaluation and imaging techniques such as magnetic resonance imaging (MRI) are utilized after specified periods to re-adjust the regiment dosages. MRI scans are generally performed after every two or three months to monitor tumor volume, progression, and its recurrence [10,15].

Thus, recent advancements in the field of cancer research emphasize employing a combination of two or more of these anti-tumor treatment plans simultaneously for tumor mitigation [9,16]. However, these procedures have adverse side effects as they employ dangerous dosages of radiation or anti-cancer drugs, which not only kill cancerous cells but are also harmful for healthy normal cells. Similarly, these treatments also hamper the effectiveness of the human body’s own immune response against cancer cells. It is hence pertinent to develop strategies for the regulation of drug and radiation dosages so that overdosing may be avoided while prioritizing human health and well-being [17].

Experimental work in the field of tumor mitigation and control is not only resource intensive but is also very time consuming [18]. Thus, extensive research has been executed to formulate mathematical models of tumor dynamics, as well as control strategies based on them [19,20]. The most basic Gompertz model considers tumor dynamics and the impact of chemotherapeutic drugs on tumor growth [21]. Similarly, refs. [21,22] considered the toxicity of anti-tumor chemo drugs on healthy and tumor cells in a three-state system model. In reality, tumor formulation in a human body triggers the immune system to destroy the cancerous cells naturally. However, this natural anti-cancer response of the human body is usually not enough to completely obliterate the tumor mass [8,23]. Thus, refs. [19,24,25] improve the tumor dynamics model by including the dynamics of human immune system response and the impact of chemotherapy on it. Similarly, ref. [1] considers a four-state brain tumor system that incorporates the healthy and immune cell dynamics into the model along with the cancerous brain cells. Ref. [16] further modified the three-state basic ODE model into a five-state system that incorporates both radiation and chemotherapeutic drugs as control inputs for tumor reduction.

Numerous control algorithms have been proposed in recent studies to halt tumor proliferation [26,27,28]. As brain tumor dynamics are nonlinear, nonlinear controllers are naturally the first choice for tumor containment through chemo and radiation dosage regulation. A fractional-order tumor system-based hybrid fuzzy sliding mode control has been proposed for immediate tumor containment [24]. Whereas ref. [29] suggested the use of a Lyapunov-based controller implemented using a two-state dynamical model. Similarly, fuzzy controllers were proposed for chemo-dose adjustment to reduce tumor advancements in refs. [25,27]. Ref. [25] also performed an elaborate performance comparison between fuzzy, state-feedback, and synergetic controllers for the application of tumor mitigation. SMC and its variants were also analyzed recently and their performances were compared with that of the supertwisting algorithm for the chemotherapy of brain tumors [28,30]. Moreover, as nonlinear controllers are generally robust, they tend to introduce oscillations or transients in system dynamics [21,25]. These transients can become harmful when left unattended, especially in sensitive applications such as tumor mitigation. Thus, incorporating a smoothing function can reduce these natural transients while maintaining the robustness of the original algorithm [28]. Similarly, optimal control theory-based optimizers have also been employed recently for tumor inhibition, yet, most of these controllers are designed based on linearized tumor dynamics [21,22,23,31]. Although chemotherapy is more effective against brain tumor progression, it is also more damaging to healthy cells. In comparison, because radiotherapy is more directed towards the location of the tumor, it is hence overall less injurious to the human body [16].

This theoretical and non-clinical study proposes an upgraded brain tumor dynamics model that incorporates the simultaneous impact of both radiotherapy and chemotherapy on tumor cells. The model also includes the dynamics of healthy and immune cells along with their interaction with the combined radio-chemotherapy dosages. Moreover, this research work proposes a novel multi-input nonlinear control-based hybrid algorithm that integrates a synergetic controller with a sigmoid function-based smoothing function to limit the damage done by these therapies to healthy and immune cells to be at a minimum. Similarly, the smoothing function provides a mechanism to mitigate any transients produced due to the action of the nonlinear controller smoothly, so that any inconvenience, for the patient under consideration, may also be minimized. Lastly, the obtained results of the proposed controllers will be analyzed after comparing them with those of recently published studies.

## 2. Materials

This study employs a mathematical framework for the analysis of brain tumor growth and the impact of combined radio-chemotherapy on it. This section outlines the state variables utilized by the fundamental model, control variables, system parameters, and research objectives, based on which the control methodology is formulated.

### 2.1. Mathematical Framework

The ordinary differential equations (ODEs)-based tumor-mathematical model utilized by this study encapsulates the intricate interactions among different cell populations and the applied treatment procedures. This model extends prior ODE-based tumor dynamics by incorporating the simultaneous impact of radiotherapy and chemotherapy [1,28]. The following state variables are considered in this model:

- T (Tumor cells): Represents a cancerous brain cell population that proliferates chaotically.
- N (Healthy cells): Represents a normal brain cell population having a direct negative impact of treatment.
- I (Immune cells): Represents the response of the immune system to the presence of tumor cells.
- R (Radiation concentration): Quantifies the amount of radiation accumulated within the body during therapy.
- C (Chemotherapy drug concentration): Quantifies the amount of an administered chemotherapeutic drug within body.

In this model, two key control inputs are regulated:

1. Radiation dosage (α): The amount of radiation applied to regulate and mitigate tumor population.
2. Chemotherapy dosage (*q*): The administered chemotherapeutic drug for tumor population inhibition.

The ODEs, characterizing complex nonlinear interactions between these state variables, along with the simultaneous impact of radio-chemotherapy, provides the necessary framework on which the proposed nonlinear controller is designed for tumor mitigation. By utilizing this comprehensive framework, an intricate balance between effective tumor subjugation, and the sustenance and proliferation of healthy cells is achieved.

### 2.2. Incorporated Dynamics

The following dynamics are considered to provide a comprehensive understanding of the system:

- Radiotherapy and Chemotherapy Effects: The model not only employs the combined impact of radio-chemotherapy on tumor mitigation, but also incorporates their adverse effects on healthy and immune cells.
- Immune System Dynamics: The immune system’s natural ability to destroy cancerous cells is integrated into the model to effectively combat the menace of cancer.
- Toxicity Reduction: By simultaneously administering a regulated radio-chemo drug dosage, the study aims to reduce the overall toxicity and health deterioration caused by the treatment to improve patient safety.

### 2.3. System Parameters

The model employs normalized parameters to describe the interactions and behaviors within the system. These parameters are crucial for simulating real-world tumor dynamics accurately. Key parameters include:

- Growth rates:
  1. $r_{1}$: Intrinsic growth rate of tumor cells.
  2. $r_{2}$: Growth rate of healthy cells.
  3. $r_{3}$: Recruitment rate of immune cells.
- Killing rates:
  1. $a_{12}$: Rate at which healthy cells eliminate tumor cells.
  2. $a_{13}$: Rate at which immune cells destroy tumor cells.
  3. $a_{21}$: Rate at which tumor cells damage healthy cells.
  4. $a_{31}$: Rate at which tumor cells weaken immune cells.
- Drug and Radiation Impacts:
  1. $N_{T}$: Proportion of tumor cells eliminated by chemotherapy.
  2. $N_{N}$: Proportion of healthy cells affected by chemotherapy.
  3. $N_{I}$: Proportion of immune cells affected by chemotherapy.
  4. ϵ: Fraction of healthy cells affected by radiation.
- Decay rates:
  1. γ: Decay rate of the chemotherapy drug dosage.
  2. σ: Decay rate of the radiation dosage.

These parameters guide the behavior of the system and ensure the realistic simulation of treatment dynamics.

### 2.4. Objective

The primary objectives of this study are as follows:

1. Efficient Tumor Mitigation: An effective reduction in the tumor population through a coordinated multi-treatment approach.
2. Diminished Side Effects: A reduction in the adverse impact of anti-tumor treatment therapy through a reduction in treatment dosages.
3. Enhanced Therapeutic Efficacy: A reduction in the overall intensity of radiation and chemotherapeutic drug dosages while robustly maintaining the considered therapeutic outcomes.

By fulfilling these objectives, the proposed framework will be able to improve patient health outcomes and enhance the safety and efficacy of brain tumor treatment.

## 3. Methods

This section provides a detailed explanation of the proposed control methodology, mathematical derivations, and simulation setup. The methods aim to achieve the study’s objectives through a novel combination of advanced nonlinear control techniques and practical implementation strategies.

### 3.1. Dynamical Nonlinear Model and System Parameters

**Figure 1:**
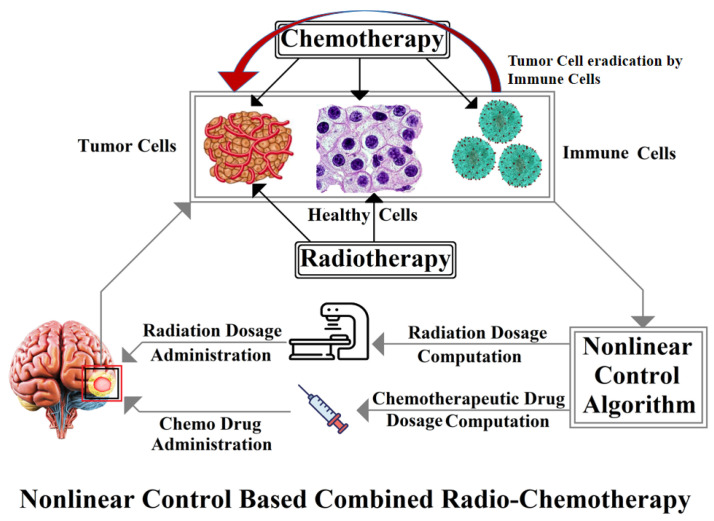
System schematic.

As discussed previously in Section 1, recent studies mostly consider the control mechanism for the regulation of chemotherapeutic drug dosage for brain tumor reduction. However, this study not only suggests a multi-therapy approach for brain tumor mitigation but it also proposes the novel sigmoid function-based smooth synergetic control. This proposed control scheme is designed based on the updated brain tumor dynamical model presented in Equation (1). The suggested model is an extension to the tumor dynamical systems presented in [1] and it considers both radiation and chemo drug dosages as control inputs. The interaction of the system state variables and the control inputs is depicted in Figure 1.


(1) $$\left\{\begin{array}{cc} \dot{T} & =r_{1}T\left(1-\frac{T}{k_{1}}\right)-a_{12}NT-a_{13}TI-N_{T}\left(1-e^{-C}\right)T-RT\\ \dot{N} & =r_{2}N\left(1-\frac{N}{k_{2}}\right)-a_{21}NT-N_{N}\left(1-e^{-C}\right)N-\epsilon RN\\ \dot{I} & =\frac{r_{3}IT}{T+k_{3}}-a_{31}IT-d_{3}I-N_{I}\left(1-e^{-C}\right)I\\ \dot{R} & =-\gamma R+\alpha \\ \dot{C} & =-\sigma C+q \end{array}\right.$$

Numerous recent studies have proposed similar nonlinear systems to analyze and explain the dynamics of brain tumors. The system of equations proposed in Equation (1) utilizes normalized parameters as proposed in refs. [16,22,25,27,32]. These parameters, with brief explanations and units of measurement, are presented in Table 1. The states “T”, “N”, and “I” in Equation (1) represent tumor cells, healthy cells and immune cells, respectively. “R” and “C” represent radiation and the quantity of the chemotherapeutic drug inside body. Lastly, “α” and “*q*” are the control inputs of the system, representing radiation and chemotherapeutic drug dosage. The intrinsic growth rates of tumor cells are higher in comparison tho those of the normal cells, indicating the rapid proliferation of tumor cells. “$r_{3}$” indicates a slower proliferation rate of immune cells, which is consistent with the tumor growth dynamics reported in the literature as tumor cells exhibit more aggressive growth dynamics in comparison to healthy and immune cells. Similarly, it can be observed that the carrying capacity of immune cells is lower than that of tumor and healthy cells, which accounts for the limited immune cell infiltration within tumor regions. Chemotherapy has a stronger impact on tumor cells in comparison to that of healthy and immune cells. Thus, the values of “$N_{T}$”, “$N_{N}$” and “$N_{I}$” are in accordance with the known side effects and efficacy of the chemotherapeutic drugs found in the literature. The relatively small fraction of normal healthy cells killed by radiation is represented by “ϵ”. In modern radiotherapy techniques, the tumor sites are targeted more precisely, resulting in this small impact of radiation on normal healthy cells. Thus, the proposed control approach is opted for with the goal of minimizing the tumor cell population (and indirectly stabilizing the healthy cell population), while applying the control signals (“α” and “q”) smoothly.

### 3.2. Proposed Control Methodology

**Figure 2:**
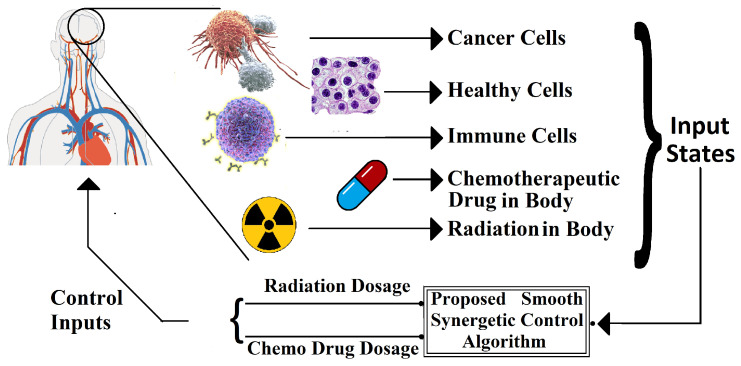
Tumor mitigation using proposed closed loop methodology.

An updated mathematical model describing brain tumor dynamics and the simultaneous impact of radiotherapy and chemotherapy on them has been utilized to formulate a smooth synergetic controller for tumor mitigation and control. The study proposes a closed loop feedback process, depicted in Figure 2, to hamper brain tumor advancements by automatically adjusting the control inputs (i.e., chemo and radiation dosages). The mathematical model that describes the brain tumor dynamics as well as their interaction with different state variables is highly complex and nonlinear. Thus, it is natural to consider nonlinear control algorithms for the containment of tumor spread. However, the nonlinear controllers are generally very robust and they tend to converge the error function to zero as fast as possible [1]. For the application of tumor mitigation using nonlinear control, this extra fast convergence rate implies that these algorithms tend to compute very high control inputs or drug dosages to decimate the tumor population swiftly [1,28]. These extra high dosages of anti-tumor therapies, however, become very problematic for the patient under consideration, as these therapies either utilize toxic drugs or harmful radiations for tumor reduction, which are very injurious to the human body [33].

**Figure 3:**
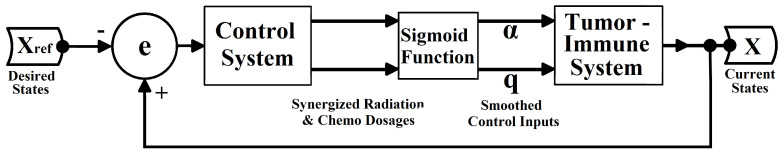
Closed loop control using SMC.

Hence, to smooth the control system’s response, this study proposes the incorporation of a smoothing function within the framework of the nonlinear synergetic controller. The synergetic control technique, being a nonlinear controller, converges the error expeditiously to zero, resulting in a very large baseline chemo dosage, as evident in [25]. The radiation and chemotherapeutic drug dosages are first computed using a standard synergetic controller. Afterwards, these computed dosages are then smoothed out using the sigmoid function. These smoothed control inputs are then supplied to the tumor dynamical model to control and regulate the radiation and chemotherapeutic drug dosages for tumor subjugation. By doing so, the rapid transitions and oscillation in control inputs may be avoided. This process of the incorporation of sigmoid-based control with synergetic control is explained and depicted in Figure 3.

Similarly, the mathematical system model presented in Equation (1) considers separate mathematical terms to represent the impact of radiotherapy and chemotherapy on system states. Thus, to design the control law to regulate radiation dosage, the state variable “C” is considered to be zero. This will remove the impact of chemotherapy from the system so that the control law related to the radiation control input may be designed separately. This process is then repeated again by considering the state variable “R” to be zero, thus removing the terms that represent the impact of radiation from the system dynamics. Hence, the control law to govern chemotherapeutic drug input is then derived independently. Hence, by considering both the control laws simultaneously, a multi-control input algorithm is designed to compute both chemo and radiation dosages simultaneously. Thus, a more efficient and effective control strategy is devised, that not only reduces the administered chemotherapeutic drug dosages by relying on radiotherapy, but also lessens the harmful impacts of chemotherapy on patients’ health and well-being.

### 3.3. Control Objectives

Although the main goal is to reduce the tumor cell population to zero, following formal control objectives, which are either related directly or indirectly to the issue fo tumor proliferation, can be deduced:

1. Tumor Cell Mitigation: The reduction of the tumor cell population (T) to zero.
2. Healthy Cell Preservation: The stabilization and perseverance of the healthy cell population.
3. Smooth Input Regulation: Used to ensure that the system control inputs “α” (radiation) and “*q*” (chemotherapy) dosages are applied smoothly to avoid any harmful transients in these toxic dosages.

### 3.4. Design of Sigmoid-Based Synergetic Controller

The main aim of the proposed anti-brain-tumor therapy is to mitigate or minimize the tumorous and cancerous cells in the brain while maximizing the number of normal or healthy cells. This can be achieved by regulating the chemo and radiation dosages simultaneously using a nonlinear synergetic controller. A synergetic controller is a unique nonlinear controller that utilizes the information of all the system states to reach a specific goal, which in this case is the mitigation of the tumor cell population. The synergetic controller is also very robust and is superior especially to the linear controllers as it can cater for system nonlinearities efficiently. Moreover, it is simpler and easier to design and implement. Yet, nonlinear controllers can introduce some transients due to their characteristic of converging the desired error to zero as swiftly as possible. This may result in the administration of excessively high dosages of radiation and chemo drugs for instant tumor mitigation. Thus, to avoid these high baseline dosages and transients, a smoothing function is proposed to smooth out any introduced or inherent transients. The proposed control loop utilizing the sigmoid function-based smoothing agent and the multi-input synergetic controller is depicted in Figure 3.

#### Control Input Derivation and Stability Analysis

As the proposed system incorporates multiple control inputs, two macro-variables will be considered, i.e., one each for the computation of radiation and chemotherapeutic drug dosages, respectively. Let:

(2) $$g_{1}=K_{1}\left(T-T_{ref}\right)+K_{2}\left(N-N_{ref}\right)+K_{3}\left(I-I_{ref}\right)+K_{4}\left(R\right)$$

(3) $$g_{1}=K_{1}\left(T-T_{ref}\right)+K_{2}\left(N-N_{ref}\right)+K_{3}\left(I-I_{ref}\right)+K_{5}\left(C\right)$$

where $K_{1}$, $K_{2}$, $K_{3}$, $K_{4}$, and $K_{5}$ are positive constants. Moreover, $T_{ref}$ and $I_{ref}$ are considered to be 0, as the goal is to reduce the tumor population to 0, and once the tumor population is reduced to 0, the immune cells must also recede accordingly. Here, the goal is to converge $g_{1}$ and $g_{2}$ to 0 with an exponential rate of convergence, which is only possible if the states successfully track their respective reference values. Consider that:

(4) $$S_{1}{\dot{g}}_{1}+g_{1}=0$$

(5) $$S_{2}{\dot{g}}_{2}+g_{2}=0$$

where $S_{1}$ and $S_{2}$ represent the rates of convergence of $g_{1}$ and $g_{2}$ to 0. Thus, both $S_{1}$ and $S_{2}$ are also positive constants. By taking the time derivative of Equation (2):

(6) $${\dot{g}}_{1}=K_{1}\dot{T}+K_{2}\dot{N}+K_{3}\dot{I}+K_{4}\dot{R}$$

(7) $${\dot{g}}_{2}=K_{1}\dot{T}+K_{2}\dot{N}+K_{3}\dot{I}+K_{4}\dot{C}$$

For simplification, consider:

(8) $$\eta =K_{1}\dot{T}+K_{2}\dot{N}+K_{3}\dot{I}$$

Thus, Equation (6) becomes:

(9) $${\dot{g}}_{1}=\eta +K_{4}\dot{R}$$

(10) $${\dot{g}}_{2}=\eta +K_{5}\dot{C}$$

The Equations (1), (4) and (9) can be utilized to work out the control law to govern the radiation dosage as:

(11) $$\alpha =\gamma R-\frac{\eta }{K_{4}}-\frac{g_{1}}{SK_{4}}$$

Similarly, Equations (1), (5) and (10) can be used to simplify the equation for the control law of chemotherapeutic drug dosage, given by:

(12) $$q=\sigma C-\frac{\eta }{K_{4}}-\frac{g_{2}}{SK_{4}}$$

Now, to prove the asymptotic stability using Lyapunov’s stability theory, consider the following Lyapunov candidate functions:

(13) $$V_{1}=\frac{1}{2}{g_{1}}^{2}$$

(14) $$V_{2}=\frac{1}{2}{g_{2}}^{2}$$

Equation (4) can be re-arranged as:

(15) $${\dot{g}}_{1}=-\frac{g_{1}}{S_{1}}$$

Similarly, Equation (5) can be re-arranged to compute ${\dot{g}}_{2}$ as:

(16) $${\dot{g}}_{2}=-\frac{g_{2}}{S_{2}}$$

By taking the time derivative of Equation (13) and simplifying using Equation (15),

(17) $$\dot{V_{1}}=g_{1}{\dot{g}}_{1}=-\frac{1}{S_{1}}{g_{1}}^{2}$$

Similarly, by taking the time derivative of Equation (14) and simplifying using Equation (16),

(18) $$\dot{V_{2}}=g_{2}{\dot{g}}_{2}=-\frac{1}{S_{2}}{g_{2}}^{2}$$

As $S_{1}$ and $S_{2}$ are positive constants, ${\dot{V}}_{1}$ and ${\dot{V}}_{2}$ become negative definite for all values of $g_{1}$ and $g_{2}$, respectively, ensuring the stability of the designed system. Equations (11) and (12) represent the synergized radiation and chemo drug inputs. In order to smooth out the response of the control system, these computed control laws are re-evaluated using a shifted sigmoid function given by:

(19) $$\alpha_{s}=\frac{2}{1+e^{-\phi_{1}\alpha }}-1$$

(20) $$q_{s}=\frac{2}{1+e^{-\phi_{2}q}}-1$$

Here, the parameters $\phi_{1}$ and $\phi_{2}$ in Equations (19) and (20) reflect the steepness of the sigmoid functions, with higher values indicating more steepness of the function.

## 4. Simulation and Results

The performance of the proposed multi-input smooth synergetic controller is evaluated in this section. The controller has been designed based on the ODE mathematical model of brain tumor dynamics presented in Equation (1).

### 4.1. Simulation Setup

Throughout the simulations, the following initial state values are considered:

$\left[T_{0};N_{0};I_{0};R_{0};C_{0}\right]$ = [0.9;0.5;0.1;0;0]. These initial conditions represents a fairly high tumor cell population amount, whereas the normal healthy cell population is kept low so that the effectiveness of the proposed control algorithm may be tested and verified more effectively.

#### 4.1.1. Environment

Simulations for this study are conducted in MATLAB (version R2016a) using the proposed nonlinear smooth synergetic control algorithm based on the nonlinear tumor dynamics presented in Equation (1).

#### 4.1.2. Controller Design Parameters

The sigmoid-based synergetic controller’s design parameters are given in Table 2. These parameters are selected to not only reduce the tumor mitigation time (settling time) as much as possible but also to make the computed control inputs smooth and transient free.

### 4.2. Performance Comparison Framework

The proposed controller is evaluated based on the following:

1. Baseline Dosage Reduction: The reduction in the initial radiation and chemotherapy dosages.
2. Faster Tumor Convergence: The time required to reduce the tumor cell population to zero.
3. Treatment Intensity Reduction: The total reduction in radiation and drug dosages over the course of treatment.

Apart from these quantitative comparison parameters, the performance of the proposed controller is also compared qualitatively with that of the recently proposed algorithms. These parameters include the following:

1. Smoothness in the applied control inputs and system dynamics;
2. The negative impact of treatment on health;
3. The ability of the controller to converge the applied inputs to zero, once the tumor population is decimated;
4. The type of therapies employed for tumor mitigation;
5. The settling time.

#### Bench-Marking

Comparative analysis between the recently proposed control algorithms and the controller proposed in this study is performed against Synergetic, state-feedback, fuzzy, super-twisting controllers and the deep reinforcement learning twin delayed deep deterministic policy gradient algorithm.

### 4.3. Results: Synergetic vs. Sigmoid Synergetic Controller

**Figure 4:**
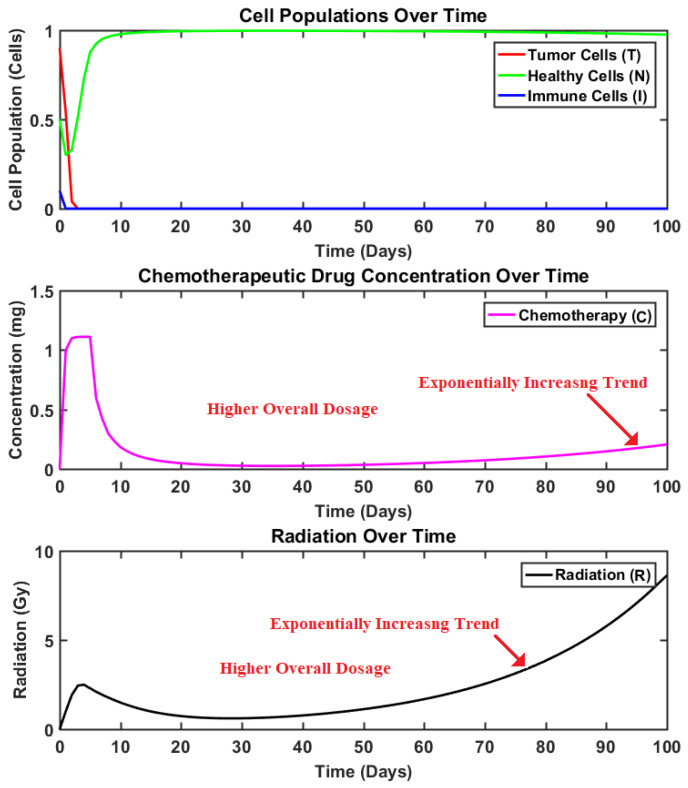
System dynamics using multi-input synergetic controller.

**Figure 5:**
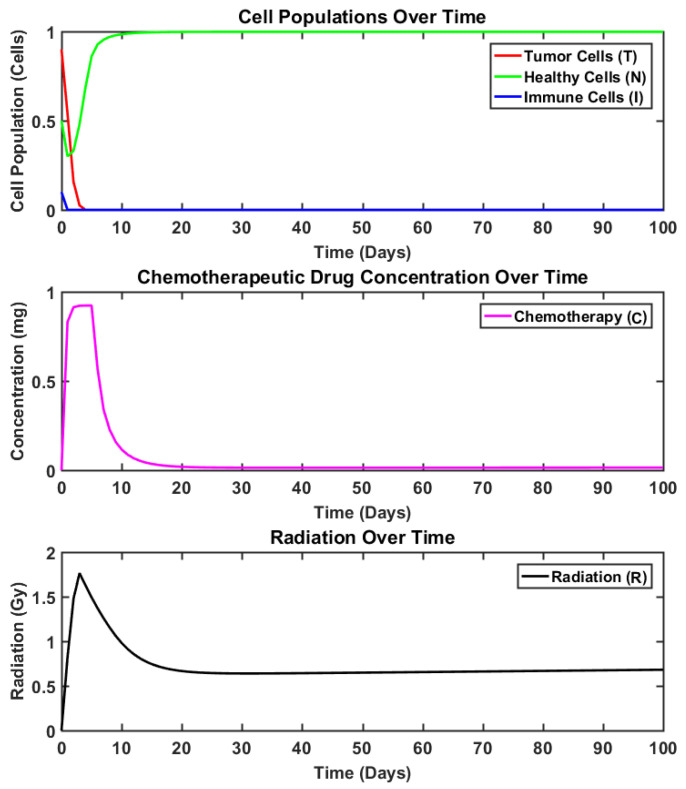
system dynamics using multi-input sigmoid function-based synergetic controller.

**Figure 6:**
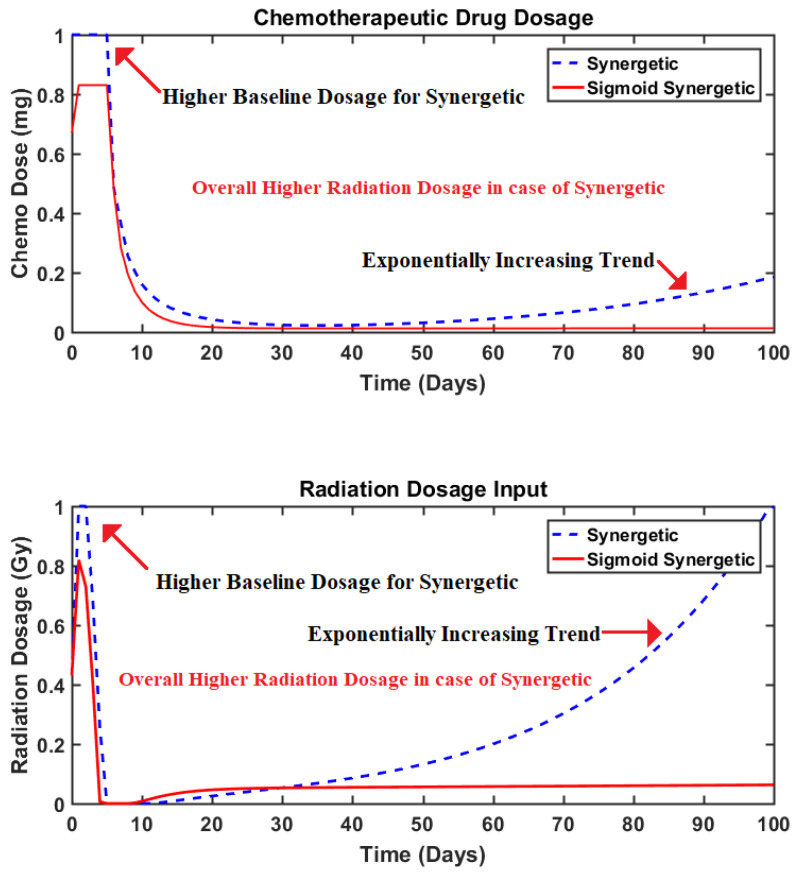
System dynamics using gradient descent-based MPC.

In order to establish the superiority and effectiveness of the proposed controller, its performance is compared with the multi-input synergetic controller. The state curves obtained using the synergetic controller for brain tumor reduction are presented in Figure 4. Meanwhile, the system response under the impact of the sigmoid-based multi-input synergetic controller is depicted in Figure 5. The curves depict the dynamics of the tumor, healthy, and immune cells. Moreover, the dynamics of the radiation and chemotherapeutic drug inputs present inside the body (represented as states R and C, respectively) at any time are depicted in the same figures, respectively. Similarly, the comparison of the computed inputs by each controller is presented in Figure 6.

Although both the controllers efficiently reduced the tumor cell population, it can be observed that the curves obtained using the synergetic controller depict higher baseline dosages of both radiation and chemotherapeutic drug inputs. Moreover, the overall dosages of both control inputs administered throughout the therapy are also higher in comparison to those of the proposed controller. Lastly, by comparing the dynamics of the states “T” and “H” of both controllers, it can be observed that their responses remain almost identical even though the dosages are notably much less in the case of the proposed controller. In fact, the action of the sigmoid-based smoothing function is more evident from Figure 6 as it stabilizes the control inputs to their steady values swiftly, making them more appropriate for such applications where drug delivery or regulation is required. As observed in Figure 4, this eventual increase in control inputs for the case of the synergetic controller thus results in a slight decrease in the population of healthy cells, as both radiation dosages and the chemotherapeutic drugs are toxins that negatively impact the healthy cells as well.

#### Chemotherapy vs. Radio-Chemotherapy

**Figure 7:**
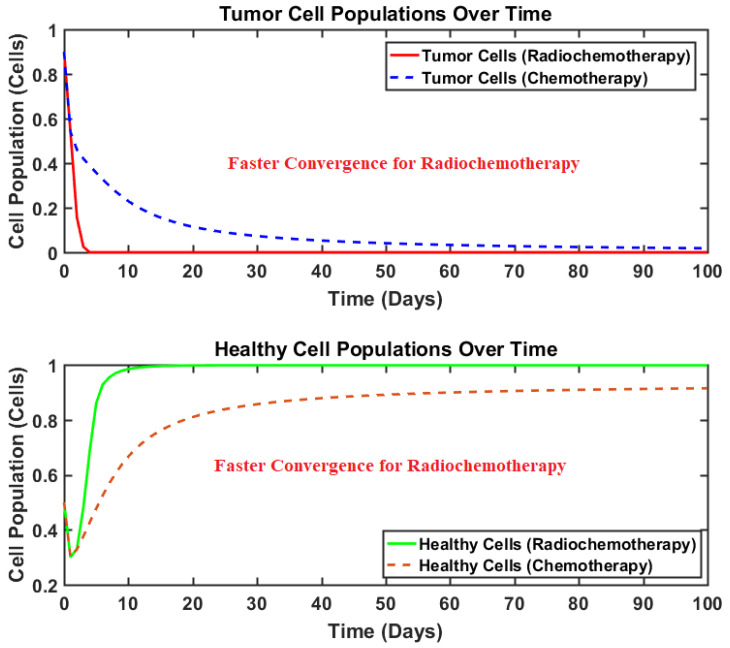
System dynamics using gradient descent-based MPC.

As discussed previously, most of the recent studies have developed and proposed only chemotherapy-based approaches for the curing of brain tumors. Since the chemotherapeutic drugs are known to cause numerous side effects that may sometimes become life-threatening as well, it is hence pertinent to reduce the dosages of chemo drugs. However, this reduction in drug dosages provides an opportunity to the cancerous cells as well. Hence, this study proposes a dual therapy approach to compensate for the reduction of the chemotherapeutic drug and its impact with that of the radiation therapy. The impact of dual radio-chemotherapy in comparison to that of only chemotherapy is presented in Figure 7.

Dual therapy is not only more potent against tumor mitigation, but it is also more efficient against tumor proliferation. The proposed dual therapy successfully mitigated the tumor population in the minimum amount of time, whereas the chemotherapy usually took a much longer amount of time to bring the tumor population to zero under similar conditions. As chemotherapy is also very lethal for healthy cells, its negative impact on the healthy cell population can be clearly observed in Figure 7. In contrast, by utilizing radio-chemotherapy, the healthy cell population recovers swiftly, proving the combined therapy to be less risky and more effective.

### 4.4. Statistical Analysis

To perform statistical analysis, the experiment performed in Section 4.3 was repeated again with slightly different initial conditions and the results were recorded. To validate the superiority, robustness and effectiveness of the proposed controller, these results are then analyzed statistically using a Mann–Whitney U-Test, Pearson’s Correlation Coefficient Test and Spearman’s Correlation Coefficient Test. Lastly, all these statistical tests are performed in MATLAB.

#### 4.4.1. Mann–Whitney U-Test—Faster Tumor Reduction Time Analysis

For the first test, the Mann–Whitney U-Test is performed by comparing the proposed simultaneous radiotherapy and chemotherapy approach with the recently proposed techniques in the literature, that are only based on chemotherapy. Group 1 shows the time taken in days by the proposed combined therapy to bring the tumor cell population to zero, whereas group 2 represents the time taken by the recently proposed algorithms in the literature to bring the tumor population to zero. The source of these values is mentioned in Table 3. The data of group 1 is recorded by performing the experiment numerous times while modifying the initial conditions of tumor and healthy cell populations from 0.9 cells per day to 0.5 cells per day for each experiment. Similarly, for group 2, the values are taken specifically from the following studies in the literature, [22,25,27,28], as these studies utilized only chemotherapy-based approaches for tumor mitigation. Moreover, these sources tested their respective proposed algorithms at different initial conditions as well. Hence, the test is performed to analyze which of the two groups suppressed the tumor population more swiftly.

group 1 = [6, 6, 6, 6, 6, 5, 4, 4, 4, 5, 5, 5, 5, 5, 4, 4, 6, 6, 6, 6, 6, 7, 7, 7, 7, 8, 8, 8, 8, 8, 9, 9, 9, 10, 10, 12]; % Radiotherapy + Chemotherapy
group 2 = [15, 20, 25, 17, 18, 20, 27, 39, 48, 45, 57, 70, 75, 78, 32, 30, 19, 18, 44, 45, 50, 58, 66, 69, 60, 68, 70, 30, 15, 71]; % Chemotherapy only

The considered null hypothesis for this statistical test is as follows:

**Hypothesis** **1 (Null Hypothesis).** *There is no significance difference between the mean time taken for the treatment between the two considered groups.*

**Results**: The following results are obtained after performing the statistical analysis:
  1. Hypothesis Test Decision (h) = 1, which means that the null hypothesis is rejected.
  2. *p*-value = $32476\times 10^{-12}<0.05$.
  3. zval = −6.9666; a large negative value indicates that group 1 took significantly less time to treat the tumor in comparison to group 2
  4. ranksum = 666.
**Significance**: The results signify that the procedure opted for the cure of group 1, i.e., combined radiation and chemotherapy took significantly less time for successful treatment in comparison to group 2, which utilized only chemotherapy for tumor suppression.

#### 4.4.2. Pearson’s Correlation Coefficient Test

The Mann–Whitney U-test validated the authenticity and efficiency of the proposed approach of combined therapy, as it significantly reduced the time of treatment. Yet, the test only considered the data of the treatment duration to reach this conclusion. Thus, to further establish the superiority of the proposed combined therapy, the Pearson correlation coefficient method is utilized that considers the treatment dosage intensities of both radiation and chemotherapeutic drug dosages along with the duration of successful treatment. The data considered to perform this statistical analysis are given below:

Radiation Dosage = [11.7, 11.66, 11.67, 11.71, 11.79, 11.94, 12.15, 12.3, 12.47, 12.72, 12.98, 13.15, 13.31, 13.56, 13.79, 13.94, 14.08, 14.34, 12.74, 13.06, 13.28, 13.5, 13.84, 14.2, 14.38, 14.57, 14.84, 15.1, 15.24, 15.37, 14.49, 13.64, 13.13, 12.64, 11.88]
Chemo Dosage = [12.3, 12.98, 12.85, 12.8, 12.778, 12.77, 12.8, 12.84, 12.88, 12.96, 13.04, 13.11, 13.17, 13.2, 13.4, 13.44, 13.5, 13.6, 12.7, 12.77, 12.82, 12.88, 12.98, 13.1, 13.17, 13.24, 13.35, 13.45, 13.51, 13.58, 13.36, 13.1, 12.96, 12.81, 12.61]
Time Taken = [6, 6, 6, 6, 6, 5, 4, 4, 4, 5, 5, 5, 5, 5, 4, 4, 5, 6, 6, 6, 6, 6, 7, 7, 7, 7, 8, 8, 8, 8, 8, 9, 9, 9, 10];

Pearson’s correlation coefficient test assumes a normal data distribution for the validation of the experiment. Thus, the Kolmogorov–Smirnov (KS) test was applied to validate the normal distribution of the collected data. Applying the KS test on the values mentioned in “Radiation Dosage” and “Chemo Dosage” resulted in *p*-values of 0.93469 and 0.73733, respectively. Similarly, for the values mentioned in “Time Taken”, the *p*-value of the KS test comes out to be 0.11232 > 0.05. All of this data is collected by conducting the experiment mentioned in Section 4.3 by varying initial conditions of tumor and healthy cell populations (as carried out in the Mann–Whitney U-test).

**Results**: The following results are obtained after performing the statistical analysis:
**Regression Coefficients:**
  1. Intercept: 36.0911;
  2. Radiation Dosage Coefficient: 1.2798;
  3. Chemo Dosage Coefficient: −3.5868.
**Model Statistics:**
  1. R-squared = 0.2236;
  2. *p*-value = 0.017432.
**Significance**: The regression model is statistically significant, with both radiation and chemotherapeutic drug dosage having a significant impact on the time taken for tumor treatment. However, chemotherapy with the negative dosage coefficient signifies that it has an overall reducing impact on the time taken for treatment. Another notable observation is the value of the intercept, which is 36.0911. Although, it appears to imply that if no radiation or chemotherapeutic drug dosage is applied, then the tumor will be subjugated on its own. However, this implication is false, because the data used to compute the intercept are recorded in case of successful treatment only. There are no data of tumor mitigation time when the chemotherapy and radiation dosages are zero. And if these dosages are kept at zero, then the tumor population will rise to the maximum amount while the healthy cell population reduces to the minimum amount. Thus, the intercept that is computed as a result of regression is providing an incorrect implication.

#### 4.4.3. Spearman’s Correlation Coefficient Test

The Mann–Whitney U-test validated the authenticity and efficiency of the proposed approach of combined therapy, as it significantly reduced the time of treatment. Yet, the Mann–Whitney U-test only considered the data of the treatment duration to reach this conclusion. Thus, to further establish the superiority of the proposed combined therapy, the Spearman’s correlation coefficient method is utilized that considers the treatment dosage intensities of both radiation and chemotherapeutic drug dosages along with the duration of successful treatment. The data considered to perform this statistical analysis are given below:

**Radiation Dosage** = [11.7, 11.66, 11.67, 11.71, 11.79, 11.94, 12.15, 12.3, 12.47, 12.72, 12.98, 13.15, 13.31, 13.56, 13.79, 13.94, 14.08, 14.34, 12.74, 13.06, 13.28, 13.5, 13.84, 14.2, 14.38, 14.57, 14.84, 15.1, 15.24, 15.37, 14.49, 13.64, 13.13, 12.64, 11.88, 199, 0, 0, 0, 0, 0, 0, 0, 0, 0, 0, 0, 0]
**Chemo Dosage** = [12.3, 12.98, 12.85, 12.8, 12.778, 12.77, 12.8, 12.84, 12.88, 12.96, 13.04, 13.11, 13.17, 13.2, 13.4, 13.44, 13.5, 13.6, 12.7, 12.77, 12.82, 12.88, 12.98, 13.1, 13.17, 13.24, 13.35, 13.45, 13.51, 13.58, 13.36, 13.1, 12.96, 12.81, 12.61, 199, 24, 14.86, 27.2, 19.73, 21.8, 24.7, 24, 14.86, 27.2, 19.73, 21.8, 24.7]
**Time Taken** = [6, 6, 6, 6, 6, 5, 4, 4, 4, 5, 5, 5, 5, 5, 4, 4, 5, 6, 6, 6, 6, 6, 7, 7, 7, 7, 8, 8, 8, 8, 8, 9, 9, 9, 10, 10, 60, 68, 78, 30, 45, 71, 60, 68, 78, 30, 45, 71];

The last 12 data points of “Radiation Dosage”, “Chemo Dosage” and “Time Taken” are collected from recently published studies in the literature, as mentioned in Table 3. The remaining set of values was obtained using the proposed algorithm by varying the initial conditions.

**Results**: The following results are obtained after performing the statistical analysis:
**Spearman Correlation:**
  1. between Radiation Dosage and Chemo Dosage: −0.3486 with *p*-value: 0.0131;
  2. between Radiation Dosage and Time Taken: −0.5316 with *p*-value: 0.0001;
  3. between Chemo Dosage and Time Taken: 0.6677 with *p*-value: 0.0000.
**Significance**: The correlation coefficient of −0.3486 indicates a monotonic relationship between “Radiation Dosage” and “Chemo Dosage”, indicating that an increase in radiation dosage results in a tendency to have a decrease in chemotherapeutic drug dosage. Similarly, the *p*-value of 0.0131<0.05 indicates that this relationship is statistically significant and shows that a higher radiation dosage potentially reduces the requirement for a higher chemotherapeutic drug dosage. The correlation coefficient of −0.5316 indicates a strong negative monotonic relationship between “Radiation Dosage” and “Time Taken”. Thus, a higher radiation dosage results in a shorter treatment time. Since the *p*-value of 0.0001 is much less than 0.05, the relationship is statistically significant. Lastly, the correlation coefficient of 0.6677 between “Chemo Dosage” and “Time Taken” indicates a strong positive monotonic relationship, suggesting that chemotherapy alone is less efficient in reducing the treatment therapy against a tumor. The *p*-value is again less than 0.5 in this case, indicating that this relationship is also statistically significant.
**Clinical Implications:** The negative correlation between the radiation and chemo dosages imply that the radiation may reduce the reliance on toxic chemotherapy in a combined treatment approach. Moreover, by integrating radiation therapy with the standard chemotherapy-based procedure, the treatment duration can be significantly reduced, making combined therapy a potentially more effective tumor-subjugating option.

## 5. Discussion: Recently Proposed Algorithms vs. Sigmoid Synergetic Controller

In order to establish the superiority and effectiveness of the proposed multi-input control algorithm, its performance is compared, both quantitatively and qualitatively, in detail with the recently proposed control algorithms in the literature.

### 5.1. Discussion: Qualitative Comparison

This section compares the performances of the recently proposed controllers qualitatively with that of the proposed sigmoid-based synergetic controller. The detailed qualitative comparison is given in Table 4. The criteria for comparison are established based on the smooth response depicted by the system dynamics, the types of therapies utilized, the negative impact on health, the ability of the controller to converge control inputs to zero after mitigating the tumor population, and the settling time (mentioned as ST). Thus, if the control action of the controller results in smooth system dynamics, then it is mentioned as “✓”. Otherwise it is marked as “×”. Similarly, If the controller has the ability to converge control inputs to zero, then it is also mentioned as ✓. However, if it fails to do so, then it is mentioned as × in Table 4. Recent studies did not consider the harmful impacts of anti-tumor therapies on human health and well-being. However, this study not only considered these impacts, but also provided an effective solution to lessen these harmful aspects of chemotherapy by regulating the control inputs and by reducing the chemo drug dosages due to the introduction of radiotherapy. Ref. [27] proposed a finite-time fuzzy logic-based controller for the reduction of cancerous cells. The proposed controller showed a bit of a sluggish response initially, resulting in an overshoot in tumor cell population. However, the controller afterwards reduced the number of tumor cells to zero within a span of 10 days. Although the settling time seems to be very low (mentioned as VL), the controller computed very high dosages of the chemo drug, making the suggested algorithm unsafe and risky. Similarly, the system dynamics also did not depict a very smooth response.

Ref. [19] also proposed a chemotherapy-based treatment scheme that combined a swarm optimization algorithm with an optimal controller for chemo drug regulation. Not only is the controller computationally very costly, but it results in the computation of extremely high drug dosages (mentioned as EH in Table 4). Thus, because of the health risk that the controller posed, it is not appropriate for the application of tumor reduction. A combined backstepping and SMC-based fuzzy logic controller has been proposed in [24], yet the study did not explicitly mention the treatment therapy utilized for tumor reduction. Moreover, the controller proposed by the study computed negative control inputs, suggesting that the drug dosage is removed from the body after administration, making it illogical and practically impossible, thus limiting the scope and practicality of the devised method. Ref. [16] also suggested combined radio-chemotherapy for tumor mitigation. The study proposed an optimal controller to do so. Yet, the controller failed to reduce the chemotherapeutic drug dosage and resulted in extremely high dosages for both radiation and the chemo drug.

Ref. [25] proposed and analyzed the performance of three nonlinear techniques, namely synergetic (marked as “a” in Table 4), state-feedback (marked as “b” in Table 4) and fuzzy logic-based controllers (marked as “c” in Table 4) for tumor reduction. Even though all three controllers computed high initial dosages and an overall high chemotherapeutic drug dosage throughout the therapy, they were unable to reduce the tumor population to zero swiftly. Moreover, the devised controllers did not consider any mechanism to quench transients produced by the nonlinear controllers. A fuzzy logic-based $P+D^{\mu }$ controller with optimized gains has been proposed in [22], using chemotherapy for cancer treatment. Moreover, the designed controller did not focus on minimizing the toxicity caused by the administered drugs. The reinforcement learning-based twin delayed deep deterministic method has been employed by [7] for tumor treatment. Although it is computationally very costly, the algorithm does not require system equations to compute drug dosages. The algorithm also resulted in high dosages of the chemo drug and is also not optimized to steadily and smoothly adjust the administered dosages. Thus, it is not convenient and is also risky for this complex application of brain tumor mitigation. Lastly, ref. [28] proposed a super-twisting algorithm for tumor mitigation, but the algorithm resulted in very high drug dosages (mentioned as VH in Table 4) as well. Moreover, the suggested algorithm computed negative control input as well, suggesting that the drug is removed from the system, which is impossible. Thus, after detailed comparison, it can be inferred that the proposed novel sigmoid-based synergetic controller clearly outperforms the other recently proposed algorithms for the application of tumor mitigation.

### 5.2. Quantitative Comparison

The algorithms proposed in the recent literature have been designed according to the requirements of numerous tumor therapies that utilize different tumor dynamics. Thus, a quantitative comparison has been presented in Table 5 so that the performance of the proposed sigmoid-based synergetic controller may be compared to other similar studies that utilize the same type of anti-tumor therapies and initial conditions. The comparison given in Table 5 considers three parameters on the basis of which the performance of the considered algorithms is evaluated. The first criterion of comparison is the initial or baseline drug or radiation dosage administered by the mentioned algorithm. This reduction in the baseline dosage is calculated in percentage by comparing it to the baseline dosages of the proposed sigmoid-based synergetic controller as depicted in Figure 6. Similarly, the second criteria for comparison is based on the time (in days) taken by the specific algorithms in comparison to that taken by the proposed sigmoid-based synergetic controller to bring the tumor cell population to zero. Lastly, the third parameter in Table 5 depicts how much reduction in treatment intensity (i.e., the total radiation and chemo drug dosage administered by the algorithm) has occurred in comparison to the other recently proposed techniques over the span of the whole treatment therapy.

As the techniques presented in studies were more focused on immediate tumor reduction, these algorithms computed maximum initial dosages. Thus, the proposed nonlinear sigmoid-based synergetic controller reduced the baseline dosage of the chemotherapeutic drug to 33% in comparison to these recent controllers. Ref. [16] also considered both radiation and chemotherapy for tumor treatment, yet the technique resulted in a high radiation dosage as well. The proposed sigmoid-based synergetic controller reduced this initial radiation dosage to 57% of that computed by the algorithm proposed in ref. [16]. Similarly, the proposed sigmoid-based synergetic controller depicts a swift convergence of the tumor cell population to zero. This is evident by analyzing the data in Table 5, as the algorithm reduced this convergence time to 60% in comparison to the other recently proposed methods. Lastly, the total dosage administered by the proposed algorithm is also the least, as it reduced the overall chemo drug dosage to 21.3%, whereas, a 93.85% reduction in radiation dosage has been observed when compared with the study mentioned in [16]. Hence, the proposed algorithm outperforms the other techniques in all aspects.

### 5.3. Challenges and Limitations in Clinical Implementation

Although the proposed multi-input sigmoid-based smooth synergetic nonlinear controller has depicted effectiveness in simulations, translating it into practical clinical practice exhibits significant limitations and challenges. These challenges include the following:

- Variation in Patient-Specific Factors: The system model presented in this study, while it is realistic, may not capture the complex and nonlinear nature of practical, real-world biological processes. Patient-specific parameters such as health conditions, genetic variations, tumor heterogeneity, etc., play a vital role in governing the response of a tumor to combined radiotherapy and chemotherapy. Tumors manifest significantly different growth and death rates, radio-sensitivity and resistance to chemotherapeutic drugs. Thus, they require a personalized model with real-time adjustments in system parameters to efficiently predict the tumor’s response to anti-tumor therapies.
- Real-Time Data Collection and Monitoring: All of the recently proposed controllers rely on frequent, accurate and precise measurements of system states, tumor size, radiation and chemotherapeutic drug dosages. Data collection in practical real-world scenarios can be very challenging and may require the use of state estimators such as a Kalman filter or nonlinear observers to effectively predict the missing data.
- Regulatory Approvals and Clinical Training: As per the guidelines for medical devices from the Food and Drug Administration (FDA) or the European Medicines Agency (EMA), the proposed controller would require extensive experimentation for its validation and compliance. Similarly, medical and healthcare professionals would require training to effectively operate the proposed controller to ensure its safety and risk-free translation from simulation to practice.

### 5.4. Clinical Aspect of Proposed Research

While our control algorithm assumes knowledge of the state variables for computation, it is important to note that the control input in our proposed algorithm is computed in units of per day. Thus, it aligns well with the practical time frame of obtaining state information. In clinical practice, the required state information (such as tumor size) can be realistically obtained through routine laboratory tests conducted at regular intervals. These periodic measurements provide a sufficiently accurate and realistic estimation of the system’s state for our control algorithm to function effectively. By operating within this framework, the proposed algorithm accommodates real-world conditions without relying on instantaneous or continuous state measurements.

#### Translation of Algorithmic Improvements into Patient Outcomes

The proposed multi-input sigmoid-based smooth synergetic controller exhibits significant improvements in terms of reduced baseline dosage, reduced tumor mitigation time, and an overall reduction in treatment intensity when compared to existing algorithms. These technical enhancements have consequential implications for patient outcomes, as discussed below:

1. Enhanced Tumor Mitigation and Control: The proposed control approach synergizes the impact of radiotherapy and chemotherapy and becomes more potent against tumor advancements in comparison to conventional single-therapy approaches. Swift tumor reduction rates have direct consequences that may result in higher survival rates and improved quality of life.
2. Shortened Treatment Duration: The controller reduces the overall treatment duration while maintaining therapeutic efficacy. This shortened treatment duration not only reduces the physical and emotional burden on the patient, but also lowers the risk of the development of treatment-related complications. Thus, the proposed approach is particularly beneficial for patients with aggressive tumor types that require immediate intervention.
3. Personalized Treatment: Based on patient-specific tumor dynamics, the proposed algorithm has the ability to dynamically adjust therapy parameters and computed dosages to ensure that the treatment is tailored to individual needs, thereby enhancing patients’ safety and survivability.
4. Reduced Toxicity: By incorporating radiotherapy into the treatment procedure, the proposed approach tends to reduce the dosage requirements and reliance on the chemotherapy. Lower chemotherapeutic drug-related toxicity has direct implications to patients’ health, safety, healthcare-related costs, and patients’ chances of survival.

## 6. Conclusions

This study proposed a novel sigmoid-based nonlinear synergetic controller to impede brain tumor progression. Moreover, this study upgraded recently proposed brain tumor dynamics into a multi-input system by incorporating the dynamics of radiotherapy along with chemotherapy for tumor mitigation and control. This updated system is then utilized to design and simulate the proposed sigmoid-based synergetic controller. The Lyapunov stability theory-based stability analysis of the designed controller has also been presented. The effectiveness of the designed controller was then validated by establishing a detailed qualitative and quantitative comparison with the algorithms and techniques proposed in recent studies. This study revealed that by combining radiotherapy with chemotherapy, the toxic impact of chemotherapeutic drugs can be sufficiently reduced. Moreover, by combining a sigmoid-based smoothing function with a synergetic controller, the system response not only became smooth and steady but it also resulted in overall lower administered dosages of radiation and chemotherapeutic drugs. Thus, by doing so, the proposed controller limits the negative impact of anti-tumor therapy and is the least detrimental for patients’ health and well-being.

## 7. Future Research Road-Map

To build upon the current study, future research directions will focus on patient-specific model adaptations by incorporating patient-specific data into the mathematical model to improve personalized treatment plans. Similarly, optimization-based algorithms will be incorporated along with nonlinear control techniques so that multi-objective approaches may be utilized to ensure minimized tumor cell population and toxicity while maintaining the robustness of the overall control algorithm. This optimization-based approach can also be integrated with reinforcement learning-based techniques, so that patient-specific parametric variations can be dynamically adjusted based on real-time patient feedback. By addressing these key issues, this study aims to bridge the gap between mathematical modeling and clinical applications so that a more effective and personalized treatment plan may be offered to cancer patients.

## Figures and Tables

**Table 1:** Normalized parameters of dynamical tumor immune system nonlinear model.

Parameter	Units and Values
$r_{1}$: Intrinsic growth rate of T	1.5 ${\mathrm{day}}^{-1}$
$r_{2}$: Intrinsic growth rate of N	1 ${\mathrm{day}}^{-1}$
$r_{3}$: Recruitment rate of I	0.01 ${\mathrm{day}}^{-1}$
$a_{12}$: Rate of killing of T by N	1 ${\mathrm{cells}}^{-1}$ ${\mathrm{day}}^{-1}$
$a_{13}$: Rate of killing of T by I	0.5 ${\mathrm{cells}}^{-1}$ ${\mathrm{day}}^{-1}$
$a_{21}$: Rate of killing of N by T	1 ${\mathrm{cells}}^{-1}$ ${\mathrm{day}}^{-1}$
$a_{31}$: Rate of killing of I by T	1 ${\mathrm{cells}}^{-1}$ ${\mathrm{day}}^{-1}$
$N_{T}$: Proportion of T eliminated by Chemo	0.5 ${\mathrm{day}}^{-1}$
$N_{N}$: Proportion of N eliminated by Chemo	0.1 ${\mathrm{day}}^{-1}$
$N_{I}$: Proportion of I eliminated by Chemo	0.2 ${\mathrm{day}}^{-1}$
ϵ: fraction of N killed by radiation	0.0008
$d_{3}$: Natural death rate of I	0.2
$k_{1}$: Carrying capacity of T	1 ${\mathrm{cells}}^{-1}$
$k_{2}$: Carrying capacity of N	1 ${\mathrm{cells}}^{-1}$
$k_{3}$: Carrying capacity of I	0.3 ${\mathrm{cells}}^{2}$
γ: Decaying rate of Chemo drug	0.082 ${\mathrm{day}}^{-1}$
σ: Decaying rate of Radiation	0.9 ${\mathrm{day}}^{-1}$

**Table 2:** Parameter values.

Parameter	Value	Parameter	Value
$K_{1}$	0.05	$S_{1}$	15.2
$K_{2}$	4	$S_{2}$	0.8
$K_{3}$	0.0005	$\phi_{1}$	9
$K_{4}$	1.62	$\phi_{2}$	2.2
$K_{5}$	0.48		

**Table 3:** Statistical analysis data sources.

Source	Baseline Dosage		Tumor Mitigation	Treatment Intensity	
	Chemo (mg)	Rad (Gy)	Time (days)	Chemo (mg)	Rad (Gy)
Optimal-Multi-Input [16]	1	1	10	199	199
Synergetic [25]	1	–	60	24	–
Statefeedback [1,25]	1	–	68	14.86	–
Fuzzy [25,27]	1	–	70	27.2	–
PID [25]	1	–	71	24.7	–
DRL TD3 [7]	3.6	–	30	19.73	–
Supertwisting [28]	1	–	15	21.8	–
Proposed Smooth-Synergetic	0.67	0.43	4	11.7	12.3

**Table 4:** Controller performance: qualitative comparison.

Source	Smooth Response	Therapy Type	Negative Impact on Health	Input Conv. to 0	ST 2% (days)
[27]	×	Chemo	High	✓	10
[19]	×	Chemo	EH	×	50
[24]	✓	N\A	N\A	✓	100
[16]	×	Chemo+Radio	EH	✓	200
[25] a	×	Chemo	High	✓	60
[25] b	×	Chemo	High	✓	68
[25] c	×	Chemo	High	✓	70
[22]	×	Chemo	High	×	20
[7]	×	Chemo	High	✓	30
[28]	✓	Chemo	VH	×	15
Sig-Syn	✓	Chemo+Radio	VL	✓	9

**Table 5:** Controller performance: quantitative comparison with proposed sigmoid SMC-based PPO.

Source	Baseline Dosage Reduction		Faster Tumor Reduction	Treatment Intensity Reduction	
	Chemo	Rad		Chemo	Rad
Optimal-Multi-Input [16]	33%	57%	60%	94.1%	93.85%
Synergetic [25]	33%	–	93.33%	50.83%	–
Statefeedback [1,25]	33%	–	94.12%	21.3%	–
Fuzzy [25,27]	33%	–	94.36%	56.3%	–
PID [25]	33%	–	94.36%	50.83%	–
DRL TD3 [7]	81.3%	–	86.67%	40.7%	–
Supertwisting [28]	33%	–	73.3%	46.36%	–

## Data Availability

The original contributions presented in this study are included in the article. Further inquiries can be directed to the corresponding author.
